# Submission

**Published:** 1890-08-02

**Authors:** 


### Words of Consolation.

### SUBMISSION.

What a difficult lesson to learn! and yet it is absolutely necessary that we should master it, whether in sickness or in health; for our happiness in this world and our hopes for the next depend on our submission to God's will. Our Blessed Lord told Saul the persecutor, when He condescended to stop that headstrong soul which was working with mistaken zeal for what it imagined to be for God's glory, "it is hard for thee to kick against the pricks." Doubtless he had had many pricks of conscience when he made havoc of the Church, and, haling men and women, committed them to prison. He was present, too, at the glorious death of the first martyr Stephen, who, with a face like an angel, gazed on the opening Heavens and with his parting breath prayed for his enemies. This constancy and the vision granted for the establishment of Stephen's faith, must have raised thoughts in Saul's heart which he found hard to resist. Yet evidently he had resisted them, and it needed for him to be struck down in his mad career, to be blind for a season, and to pass that season in fasting and prayer, ere he could understand that he must serve the God whom he loved in that God's own way. From that time forward Paul suffered with some grievous bodily infirmity, a thorn in the flesh as he himself calls it, which, notwithstanding his thrice preferred earnest prayer for its removal, was never taken away. The answer he received was "My grace is sufficient for thee: for my strength is made perfect in weakness." And so he went on taking pleasure in infirmities, in reproaches, in necessities, in persecutions, in distresses for Christ's sake, till, as Paul the aged, he could say, "I have fought a good fight, I have finished my course, I have kept the faith; henceforth there is laid up for me a crown of righteousness which the Lord shall give me at that day."

All these things are written for our admonition and learning. The Lord's hand is not shortened. He will interpose for us as He did for Saul of Tarsus, though we are going on resolutely in our own way, determining to choose our own path in life, probably striving hard in the wrong road, absolutely sure we can do better for ourselves than our Heavenly Father can do for us. How active we could be in His service with health and strength, we think! how good and holy if we had not those tempers and oppositions in other people to contend with! A life without cares and troubles and sickness, that is the journey we map out for ourselves. Where it will end we do not stop to inquire; if we did we should most likely see, in idleness, self-indulgence, and a hard heart. Happy for us when God steps in with warning, with affliction, with correction, and we ask, with the convinced and repentant Paul, Lord, what wouldst Thou have us to do? Many a humble and contrite heart echoes the words of the poet—

> I was not ever thus, nor prayed that Thou
> Shouldst lead me on;
> I loved to chose and see my path; but now
> Lead Thou me on.
> I loved the garish day, and, spite of fears,
> Pride ruled my will: remember not past years.

What a sad sight do those past years recall! Struggles and rebellion at the chastening rod, and a tighter grasp of those things on which we had set our hearts. But the precious balms have broken the heads of our pride and self-will.

